# Modeling the Performance of Full-Scale Anaerobic Biochemical System Treating Deinking Pulp Wastewater Based on Modified Anaerobic Digestion Model No. 1

**DOI:** 10.3389/fmicb.2021.755398

**Published:** 2021-09-21

**Authors:** Yifeng Huang, Yongwen Ma, Jinquan Wan, Yan Wang

**Affiliations:** ^1^School of Environment and Energy, South China University of Technology, Guangzhou Higher Education Mega Center, Guangzhou, China; ^2^Sino-Singapore International Joint Research Institute, Guangzhou, China; ^3^Guangdong Plant Fiber High-Valued Cleaning Utilization Engineering Technology Research Center, Guangzhou, China

**Keywords:** anaerobic digestion, deinking pulp wastewater, full-scale anaerobic reactor, Anaerobic Digestion Model No. 1 (ADM1), anaerobic biochemical treatment

## Abstract

The deinking pulp (DIP) is a main resource for paper making, and the wastewater from DIP process needs to be treated. Anaerobic biochemical technique has been widely applied in DIP wastewater treatment, due to the remarkable capability in reducing high chemical oxygen demand (COD). In this study, a mathematical simulation model was established to investigate the performance of a full-scale anaerobic biochemical system for treating DIP wastewater. The model was based on Anaerobic Digestion Model No. 1 (ADM1), which was modified according to the specific anaerobic digestion process for DIP wastewater treatment. The hydrodynamic behavior of a full-scale anaerobic biochemical system was considered in this model. The characteristics of the influent DIP wastewater were assessed, and then, the substrate COD proportion was divided successfully for the necessity of ADM1 applying. The Monte Carlo technique was implemented to distinguish the most sensitive parameters that influenced the model output indicators comprising effluent COD and biogas production. The sensitive parameters were estimated and optimized. The optimized value of *k*__m_pro_ is 12.02, *K*__S_pro_ is 0.35, *k*__m_ac_ is 4.26, *K*__S_ac_ is 0.26, *k*__m_h2_ is 16.62, and *K*__S_h2_ is 3.21 × 10^–5^. The model was calibrated with 150 days operation values measured in the field. The subsequent 100 days on-site values were used to validate the model, and the results obtained by the simulations were in good agreement. This study provides a meaningful and theoretical model guidance for full-scale wastewater anaerobic biochemical treatment simulation.

## Introduction

Due to the shortage of raw fiber from wood, wastepaper recycling has become an important source of pulp and paper production (Saxena and Singh Chauhan, 2017; Ozgun, 2019). The deinking pulp (DIP) process is an essential component of wastepaper recycling, which involves the removal of ink from printed paper (Xu et al., 2011). Large amounts of wastewater are inevitably produced by the deinking process, which needs to be treated carefully (Song et al., 2018). The amount of wastewater produced by pulp and wastepaper treatment was estimated to have grown by 60% from 2012 to 2020 (Meyer and Edwards, 2014). It was estimated that 400 million tons of paper were produced annually, and the wastewater is 10∼100 m^3^ per ton of pulp and paper production (Irizar et al., 2018). Various strategies such as flotation, sedimentation, filtration, and aerobic activated sludge have been utilized to DIP wastewater treatment (Simstich et al., 2012; Irizar et al., 2018). In last two decades, the application of anaerobic digestion (AD) to the treatment of wastewater with high organic contents has grown rapidly, including wastewater produced from pulp and paper making. AD is a biological process that involves the transformation of organic compounds into biogas under an oxygen-free state, where the reduction in the chemical oxygen demand (COD) is up to 80% (Buzzini and Pires, 2002). In addition, the biogas, a byproduct from AD process, is a profitable green fuel (Kamali et al., 2016; Wu et al., 2019, 2020).

Previous researches showed that, because of the difficulty of biochemical degradation or decomposition of ingredients in high organic wastewater, pre-acidification process was employed to promote biodegradability with a short hydraulic residence time (HRT) in anoxia state (Çalışkan and Azbar, 2017; Diamantis and Aivasidis, 2018; Wu et al., 2020). After pre-acidification, the high organic wastewater will always be treated by the anaerobic biochemical reactor, which plays a key role in the removal of COD concentration (Sarathai et al., 2010). The internal circulation (IC) reactor is a specific representative type of high-rate anaerobic biochemical reactor, which can be viewed as two upflow anaerobic sludge blanket (UASB) reactors stacked together. In contrast to the 15 kg COD m^–3^ d^–1^ loading of UASB, the organic loading rate capacity of IC is up to 20–50 kg COD m^–3^ d^–1^ (Rajagopal et al., 2013; Karadag et al., 2015; Wang et al., 2015; Hamza et al., 2016). The IC reactor has been widely utilized in industrial wastewater anaerobic treatment, such as beer production (Chen et al., 2021), cotton pulp manufacturing (Cui et al., 2011), food processing (Guo et al., 2018), and paper making (Irizar et al., 2018), etc.

Mathematical models have been applied broadly in wastewater treatment for simulation (Feldman et al., 2017), design (Flores-Alsina et al., 2012), supervision (Rodriguez-Roda et al., 2002), optimization (Rivas et al., 2008), and even benchmark control (Gernaey et al., 2014). Compared with aerobic biochemical process, AD process is much more complicated and often easily disrupted by several adverse factors, for example, organic overload, acute temperature variance, or the presence of inhibitory substances, etc. For these reasons, the wastewater plants have to adopt a larger reactor, or to utilize on-site monitoring device. But the larger reactor needs more cost, and the lack of reliable, unfeasible and cost saving limits the application of on-site device. Therefore, AD models have been used to indirectly provide profitable information about the operation condition of the reactor (Irizar et al., 2018). Several types of models have been proposed for describing the details of the AD process (Diez Blanco et al., 1995; Chen et al., 2016). In particular, Anaerobic Digestion Model No. 1 (ADM1) published by the International Water Association (IWA) task group in 2002 (Batstone et al., 2002), has expanded rapidly to even became a *de facto* standard model for AD simulation (Lier et al., 2015). ADM1 is a structural model that comprises 19 steps for defining biochemical processes, where physico-chemical equations are used to describe ion association/dissociation and gas–liquid phase transfer (Batstone et al., 2015; Rubio et al., 2020).

The originally reported example of the application of ADM1 involved a completely stirring tank with a constant volume and unidirectional influent–effluent stream (Batstone et al., 2002). Most of previous studies that used ADM1 were conducted at the lab-scale, where it was assumed that the ingredients were uniformly or homogeneously distributed in the AD devices (Naessens et al., 2012; Yu et al., 2012; Van Hulle et al., 2014; Chen et al., 2015; Poblete et al., 2020). The hydraulic behavior of these reactors could be simplified as complete stirring because of their small size (Lauwers et al., 2013). However, Batstone et al. (2005) found a scaling effect between a lab-scale UASB reactor and a full-scale one. Van Hulle et al. (2014) stated that a corrected description of the mixing behavior of a full-scale AD reactor is required to simulate the reactor’s performance in an appropriate manner. An incorrect description of the hydraulic behavior of a full-scale reactor would lead to over-calibration of the stoichiometric and kinetic parameters of the model, thereby affecting the experimental results (Uggetti et al., 2010; Liotta et al., 2015). Consequently, the hydraulic behavior of the full-scale AD reactor should be considered when ADM1 is applied for simulation. As far as now, most previous applications of ADM1 focused on lab-scale studies and few have considered full plant-wide application of ADM1.

In the present study, we aimed to develop a simulation method to model the performance of an anaerobic biochemical system treating DIP wastewater. The system comprised an anaerobic pre-acidification tank connected to a full-scale IC reactor at a pulp and paper mill that is located in Guangzhou City (Guangdong Province, China; Supplementary Figure 1). The model of pre-acidification process has few been fully research.

According to the running status of this anaerobic biochemical system and some previous researches (Feldman et al., 2017, 2019; Irizar et al., 2018), a single continuous stirred tank reactor (CSTR) was used to represent the hydrodynamics of the pre-acidification tank and a series of CSTRs was applied to represent that of the full-scale IC reactor. ADM1 was integrated with these hydrodynamic models in the simulation method, which we utilized to simulate the reduction of the COD content and biogas production in this system. Furthermore, components of the influent DIP wastewater were classified based on the theoretical assumptions of the IWA task group. The results showed that this model was effective at simulating the performance of a full plant-wide anaerobic biochemical system treating wastewater. As far as we know, no similar study has been published previously.

## Materials and Methods

### Overview of Deinking Pulp Wastewater Treatment Plant

The DIP process of this pulp and paper mill is based on a commonly used chemical deinking technique. According to the actual production condition, a maximum 20,000 m^3^ day^–1^ of high-concentration DIP wastewater need to be treated, the COD of which is over 2000 mg L^–1^.

The DIP wastewater treatment plant has four main sections. Firstly, large particulate pollutants are removed from the DIP wastewater in the physical pre-treatment section. Next, fine particles and dissolved pollutants are absorbed, converted, and reduced in the anaerobic biochemical treatment section that includes an anaerobic pre-acidification tank and an anaerobic biochemical IC reactor. The COD content of the DIP wastewater is mostly converted and degraded there. Then, the wastewater enters an aerobic treatment system section followed by an advanced oxidation section for further treatment. Finally, the wastewater satisfies the local effluent standard and it is discharged.

### Deinking Pulp Wastewater Quality

This mill utilizes recycled wasted newspapers and magazines, accounted for about 80 and 20%, respectively, to produce high-end newsprint. The raw DIP wastewater has to been purified in DIP wastewater treatment plant. The main quality indicators for the raw DIP wastewater are shown in Supplementary Table 1.

After filtering and settling in the physical pre-treatment section, large particles such as shredded paper, large fiber particles, inorganic filler blocks, and residual ink are removed from the raw DIP wastewater. After that, the DIP wastewater that enters the anaerobic biochemical treatment section mainly comprises fine fiber particles, soluble cellulose (solubilized fiber), surfactants (fatty acid organics from deinking agents), and fine ink particles. The color of the wastewater is grayish yellow at this stage. Through long-term observations, the effluent water quality indicators after anaerobic biochemical system treatment are listed in Supplementary Table 2.

### Wastewater Characterization

The main application objective of ADM1 developed by IWA is to model the AD process for waste activated sludge from sewage treatment plant. The input variables in the model are related to the compositions of the particulate materials. Therefore, two crucial issues must be addressed when using ADM1 to simulate the AD treatment of various types of substrates or organic wastewater. The first one is how to separate and classify the inflow components, where the input variables for the model must be determined according to the inflow substrates. The other one is how to select the values for the parameter set, where it is first necessary to estimate the sensitive kinetic parameters in the model, before then to calibrate them (Kleerebezem and Van Loosdrecht, 2006; Girault et al., 2012). In the following, we describe the theoretical assumptions of ADM1, as well as the separation and classification of the influent substrates in DIP wastewater. These classification assumptions and methods of inflow components are given in Supplementary SI1.

In the actual wastewater treatment process, the wastewater components are not consistent at all times. The pollutant components may differ within a certain range according to the variations in the mill’s production status. Clearly, none of the assessment methods described above can provide real-time information regarding the wastewater components. In particular, the component interpretation method might not be capable of completely and accurately classifying the COD components. However, the average measurement results obtained based on multiple samples can be used to represent the actual composition. In addition, the component interpretation method is relatively simple to conduct and the detection results can be applied to determine the contents of different components, before classifying and interpreting the COD components of the reactor influent. For these reasons, the component interpretation method was applied in the present study.

### Model Development

According to ADM1 released by IWA, the AD processes involve disintegration, hydrolysis, acidogenesis, acetogenesis, and methanogenesis (Batstone et al., 2002). Among these stages, disintegration and hydrolysis are extracellular processes conducted by bacteria. The actions of extracellular enzymes decompose macromolecular organic matter into proteins, carbohydrates, and lipids, which are subsequently hydrolyzed into monosaccharides, amino acids, long-chain fatty acids (LCFAs), and other molecules. Monosaccharides and amino are absorbed in the acidogenesis step, and then converted into VFAs and hydrogen. Subsequently, LCFA and VFAs are transformed into acetate at the acetogenesis step. Finally, acetate and hydrogen are transformed into CH_4_ or CO_2_ in methanogenesis step (Supplementary Figure 2).

#### Development of the Anaerobic Pre-acidification Tank Model

Previous research and experience of wastewater engineering have shown that pre-hydrolysis and pre-acidification before fully anaerobic biochemical treatment are beneficial of materials such as industrial organic wastewater derived from food, tanning, pulp, printing, dyeing, and pharmaceutical production (Ahn et al., 2001; Diamantis and Aivasidis, 2018; Al-Rubaye et al., 2020; Wu et al., 2020). Because an anaerobic or facultative pre-treatment section with a relatively short HRT, before subsequent fully anaerobic treatment, can enhance the biodegradability of wastewater. The explanation for anaerobic biological mechanism of pre-hydrolysis (pre-acidification) is provided in Supplementary SI2.

The maximum volume of the pre-acidification tank in used is 2250 m^3^. The tank is equipped with a hyperboloid mixer, which forms a continuous fully mixed interior hydrodynamic state. The DIP wastewater is acidified for almost 2 h here. Due to the short HRT and hydraulic state, the retention of methanogenic bacteria and accumulation of bacteria do not occur in the tank. In general, methanogenesis does not occur in the tank. As seen in Supplementary Figure 2, the anaerobic biochemical processes happening in pre-acidification tank involve disintegration, hydrolysis, acidogenesis, and acetogenesis. In addition, according to ADM1 released by IWA, the equations for modeling the biochemical reactions in the pre-acidification tank are expressed as follows:


(1) $$\frac{d\,S_{i,a}}{d\,t}=\frac{Q_{i\,n\,f}}{V_{a}}\,\left(S_{i,i\,n}-S_{i,a}\right)+\sum_{k=1}^{15}\upsilon_{i,k}\,\rho_{k}$$

(2) $$\frac{d\,X_{i,a}}{d\,t}=\frac{Q_{i\,n\,f}}{V_{a}}\,\left(X_{i,i\,n}-X_{i,a}\right)+\sum_{k=1}^{15}\upsilon_{i,k}\,\rho_{k}$$

Where $\frac{d\,S_{i,a}}{d\,t}\,\mathrm{and}\,\frac{d\,X_{i,a}}{d\,t}$ represent the time derivatives of the soluble and particulate substrates in the tank, respectively, *Q*_*inf*_ is the feed flow rate (m^3^ day^–1^), *V*_*a*_ is the pre-acidification tank volume (*V*_*a*_ = 750 m^3^), *S*_*i*,*in*_*andX*_*i*,*in*_ are the soluble and particulate substrates in the feed flow (kgCOD m^–3^), and *S*_*i*,*a*_*andX*_*i*,*a*_ represent the soluble and particulate substrates in the tank (kgCOD m^–3^). Moreover, the term $\sum_{k=1}^{15}\upsilon_{i,k}\,\rho_{k}$ represents the sum of the kinetic rates for process *k* multiplied by the rate coefficients (*ν_*i,k*_*). It is assumed that methanogenesis does not occur in the pre-acidification tank, so all of the processes proposed by IWA are involved, except for the uptake of acetate and hydrogen, and the decay of aceticlastic methanogens (*X*_*ac*_) and hydrogen-utilizing methanogens (*X*_*h2*_). These uptake and decay processes are considered to be related to methanogenesis.

#### Development of the Internal Circulation Reactor Simulation Model

Chen et al. (2021) who analyzed a full-scale IC reactor treating brewery wastewater, found that the bacterial community was significantly different at the diverse layers. The fermentation and acidification were mainly accomplished at bottom layer, but methane production was achieved at upper and middle layers. Recent studies (Feldman et al., 2017; Irizar et al., 2018) divided the hydrodynamics of the full-scale IC reactor into three CSTRs in series.

**FIGURE 1:**
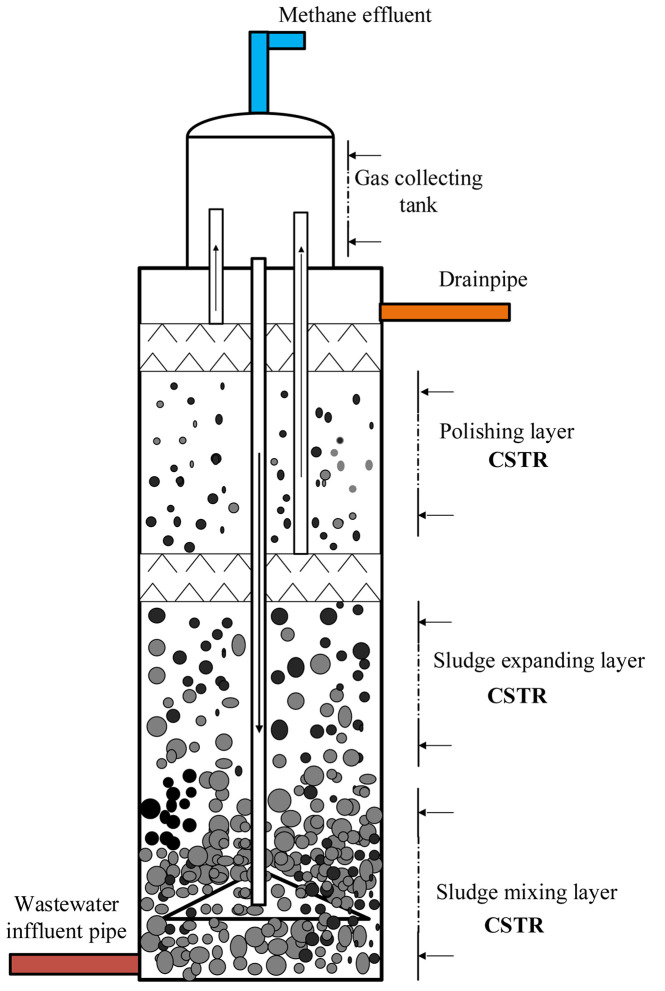
Schematic diagram of three CSTRs in series simulating hydrodynamic of full-scale IC reactor.

The size (height and diameter) of the full-scale IC reactor under researched is φ 24 m × 12.5 m. The design volume is 2900 m^3^, and the active volume is almost 2250 m^3^. The wastewater would retain approximately 8 h for anaerobic treating. Hence, we divided this reactor into three layers and CSTRs in series based on the internal state of the reactor. The bottom of the reactor retains large sludge granules. The sludge granules expand and fluidize in the middle part, and a small amount of floating sludge is present at the top part. The internally generated biogas is separated by gas–liquid–sludge separators and then collected by a gas collection tank in the headspace. The internal states of the reactor and three CSTRs in series in the separate models are illustrated in Figure 1.

The full ADM1 released by IWA was applied to simulate the biochemical reaction for each CSTR. The reaction equations for each CSTR and the diffusion equation for the gas phase under constant volume of gas in the gas collection tank, respectively, are given as follows:


(3) $$\frac{d\,S_{i.c}}{d\,t}=\frac{Q_{i\,n\,f}}{V_{c}}\,\left(S_{i,i\,n}-S_{i,c}\right)+\sum_{i=1}^{19}\upsilon_{i,j}\,\rho_{j}+\mathrm{transport}\,\mathrm{terms}$$

(4) $$\frac{d\,X_{i},c}{d\,t}=\frac{Q_{i\,n\,f}}{V_{c}}\,X_{i,i\,n}-\frac{X_{i,c}}{t_{r\,e\,s,X}+\frac{V_{c}}{Q_{i\,n\,f}}}+\sum_{i=1}^{19}\upsilon_{i,j}\,\rho_{j}$$

(5) $$\frac{d\,S_{g\,a\,s,i}}{d\,t}=-\frac{S_{g\,a\,s,i}\,Q_{g\,a\,s}}{V_{g\,a\,s}}+\rho_{T,i}\,\frac{V_{c}}{V_{g\,a\,s}},$$

Where $\frac{d\,S_{i,c}}{d\,t}\,\mathrm{and}\,\frac{d\,X_{i,c}}{d\,t}$ represent the time derivatives of the soluble substrates and particulate substrates for each CSTR part in the reactor, respectively, *Q*_*inf*_ is the feed flow rate (m^3^ d^–1^), *V*_*c*_ is the volume of each CSTR (*V*_*c*_ = 750 m^3^, the whole reactor volume is 2250 m^3^), *S*_*i*,*in*_*andX*_*i*,*in*_ are the soluble substrates and particulate substrates in the feed flow (kgCOD m^–3^), and *S*_*i*,*c*_*andX*_*i*,*c*_ represent the soluble substrates and particulate substrates in each CSTR (kgCOD m^–3^). The term $\sum_{j=1}^{19}\upsilon_{i,j}\,\rho_{j}$ represents the sum of the kinetic rates for process *j* multiplied by the rate coefficients (*ν_*i,j*_*). In addition, *t*_*res*_,_*X*_ denotes the extended retention of solids such that Sludge Retention Time (SRT) is set above HRT (*t*_*res,X*_ = 40 days) (Batstone et al., 2002). The transport term is related to dissolved insoluble gasses(*S*_*gas*,*i*_), such as carbon dioxide, methane, and hydrogen transferring into the liquid phase. *ρ_*T,i*_* is the specific mass transfer rate of gas i at temperature *T*. *V*_*gas*_ represents the volume of the gas collection tank (*V*_*gas*_ = 250 m^3^). *Q*_*gas*_is the gas flow (N m^3^ day^–1^).

#### Development of the Whole Anaerobic Biochemical System Model

The overall anaerobic biochemical system treating DIP wastewater, considered in this study, includes a pre-acidification tank and full-scale IC reactor (Supplementary Figure 1). The models developed for the tank and the reactor were built as isolated modules. Each module included all of the details described above and it was implemented using Matlab 2017b/Simulink. The simulation model for the whole anaerobic biochemical system combined the two separate modules as a single unit.

The simulated influent “DIP wastewater” first entered the pre-acidification tank module to undergo acidification. The “acidified wastewater” then entered the full-scale IC reactor module for full anaerobic treatment. The simulated inflow rate was based on the measured wastewater discharge rate, which varied according to the actual production status. The volume of the tank relative to that of the reactor was a ratio of 1:2. Thus, the HRT ratio was also 1:2 under the same inflow rate. Using feed pump control, the HRTs for the tank and the reactor were controlled to about 4 and 8 h, respectively. These inflow rates were also implemented in the combined simulation module for the whole AD system.

### Parameters Identification

Anaerobic Digestion Model No. 1 is a complex mathematical model with numerous parameters, including stoichiometric parameters, physicochemical parameters, and biochemical parameters. All of these parameters affect the model’s output but the sensitivities of parameters related to this output may vary dramatically from one to another. Sensitivity analysis has been used widely to identify significant parameters with the greatest effects on the outputs of models (Bernard et al., 2001). The approaches used to identify sensitive parameters depend on local sensitivity analysis or global one.

Most previous AD simulation studies were based on lab-scale experiments. The local sensitivity analysis method was usually applied in these studies (Tartakovsky et al., 2008; Barrera et al., 2015; Li et al., 2020). This method generally involves analyzing the different outputs obtained when an individual parameter is varied over a defined range while the other parameters remain constant. However, this method generates a linear regression equation that only represents the response of the model for a set of given points, and thus it cannot provide effective details of the correlations or aggregation errors among the various parameters (Donoso-Bravo et al., 2011).

The actual full-scale anaerobic plant reactor process is much more complicated than the lab-scale process. The lab-scale experimental condition can be precisely defined or controlled. But the characteristics of a full-scale reactor are frequently dependent on multiple factors, such as flow rate, water ingredient, temperature, which are nonlinear, time variant, and uncontrollable. The global sensitivity analysis method can cover the entire domain of the model and provide more comprehensive analysis results, thereby overcoming the deficiencies of the local sensitivity analysis method. As a result, the global sensitivity analysis method needs to be applied when modeling a full-scale anaerobic reactor. As a global sensitivity method, the Monte Carlo technique was implemented in this study. Monte Carlo technique is a mathematical method, which uses a set of representative global samples to investigate the entire model space. Monte Carlo algorithms tend to be simple, flexible, and scalable, and can reduce complex models to a set of basic events and interactions (Kroese et al., 2014). Monte Carlo technique is suitable for model analysis of full-scale anaerobic reactor.

### Model Calibration and Validation Implementation

The proposed anaerobic simulation model was calibrated and validated using real samples obtained from the pulp and paper mill anaerobic treatment system. An operating period of 250 days was selected to check the model, where samples from the first 150 days were used to optimize the sensitive parameters and calibrate the model, and samples from the next 100 days were employed to validate the effectiveness of the model. The set of differential and algebraic equations in ADM1 was implemented using Matlab 2017b/Simulink with ODE45s solvers, as recommended by Rosen and Jeppsson (2006).

### Sample Collection and Analysis

Water samples were taken from influence and effluence of the pre-acidification tank and full-scale IC reactor, respectively, every day. The COD concentration of the water samples was determined using the potassium dichromate method. The composition of untreated DIP wastewater was taken every week, and then sampled and analyzed according to standard methods (American Public Health Association, 2005), and the results are presented in Table 1. The flow rate of the biogas from the full-scale IC reactor, was acquired from the biogas flowmeter installed in methane treatment system.

**TABLE 1:** The main component content and COD concentration conversion of DIP wastewater.

	Mass concentration mg L^–1^	Converted to COD concentration kgCOD m^–3^
Total COD	–	934.98 ± 63.29
Soluble COD	–	862.84 ± 72.99
Soluble monosaccharide (S_*su*_)	354.28 ± 54.56	425.14 ± 65.47
VFAs (accounted as S_ac_)	184.88 ± 40.19	197.82 ± 42.89
Anionic surfactants (accounted as stearic acid, X_li_)	19.24 ± 6.32	51.77 ± 14.71
Inert soluble (S_I_)	–	197.87 ± 19.47
ssCOD (accounted as carbohydrate, X_ch_)	–	72.14 ± 19.71

## Results and Discussion

### Influent Chemical Oxygen Demand Classification

The characteristics of the DIP wastewater that influenced the anaerobic biochemical treatment section were determined by gas chromatography-mass spectrometry. Many organic compounds that comprised the soluble COD (SCOD) were detected in the wastewater solute samples. The main compounds included polysaccharides, VFAs, and surfactants (surface active agents). The temperature of the DIP wastewater discharged from the DIP workshop usually ranged from 40 to 50°C. At this relatively high temperature, the cellulose and hemicellulose in the fine fibers obtained from wastepaper were hydrolyzed into soluble polysaccharides. The polysaccharides were then decomposed into VFAs or other small molecules such as organic acids. Due to the requirements of the DIP technique, industrial soap (mainly sodium stearate) is used as a deinking agent in the deinking process, thereby explaining why surfactants were detected. The trace amounts of soluble protein detected in the water samples indicated that microorganisms participated in the degradation of fine fibers during the transport of wastewater from the DIP workshop. The DIP wastewater was sampled and analyzed, the results of which are presented in Table 1.

The composition of DIP wastewater is very complicated. According to the ADM1 requirements and the detection results of DIP wastewater, also considering the flexibility of the model, the influence components of the COD distribution were simplified as followed. The carbohydrates obtained from the degradation of fibers were regarded as monosaccharides (S_su_) and they accounted for about 49% of the influent SCOD. VFAs were calculated as acetic acid (S_ac_) and they accounted for about 22% of the influent SCOD. Surfactants from the DIP workshop were regarded as sodium stearate (lipid X_li_) and they accounted for about 6% of the soluble SCOD. According to the wastewater quality characterization method proposed by Ekama et al. (1986), 90% of the effluent COD from the subsequent aerobic system was regarded as an inert soluble component (S_I_; not degradable by microorganisms) which accounted for about 23% of the influent SCOD. The effect of the suspended solids COD (ssCOD) was equal to the difference between the total COD (TCOD) and SCOD, which accounted for 7% of the influent TCOD, and it was regarded as the particulate carbohydrates X_ch_. The proportion of the COD composition is given in Supplementary Figure 3.

### Sensitivity Analysis Results

**FIGURE 2:**
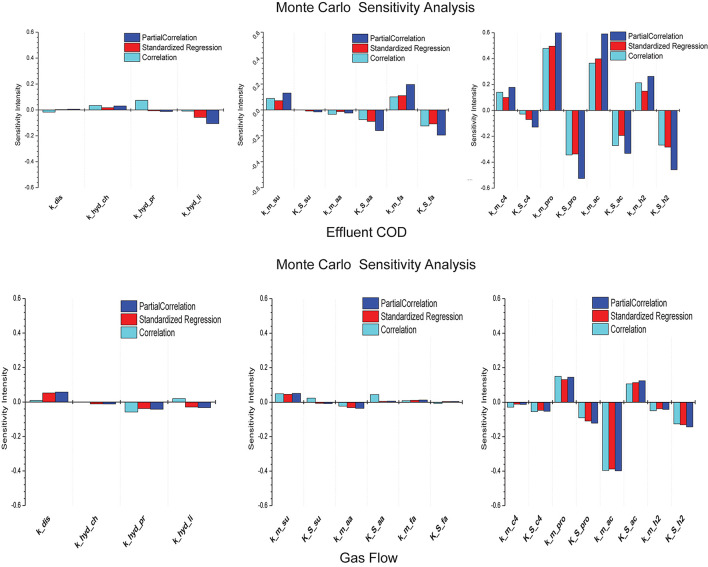
Schematic of sensitivity analysis result of 18 kinetic parameters relating to COD*_*eff*_* and biogas flow of AD system. Monte Carlo of Partial Correlation, Standardized Regression and Correlation for every parameter were respectively implemented in the sensitivity analysis.

In total, 18 kinetic parameters are employed in ADM1. The effluent COD (COD*_*eff*_*) and biogas production by the AD reactor are usually the main issues considered for paper and pulp mills. Therefore, our proposed model of this anaerobic biochemical system was used to simulate the changes in COD*_*eff*_* and biogas production. A random set of parameters was generated for evaluation by using the Monte Carlo method for the model specifications. This set comprised 500 random parameter pairs. The initial values of the parameters were the values recommended by IWA in order to simulate a medium temperature and high rate reactor. Histograms illustrating the partial correlations, standardized regression, and correlations according to the sensitivity analysis evaluation results are shown in Figure 2.

Figures 2A–C show that the Monod absorption rates for propionate (*k*__m_pro_), acetate (*k*__m_ac_), and hydrogen (*k*_m_h2_) had strong positive correlations with COD*_*eff*_* for the anaerobic biochemical system, whereas the half-saturation constants of these parameters (*K*__S_pro_, *K*__S_ac_, and *K*__S_h2_) had significant negative correlations with COD*_*eff*_*. In addition, the Monod absorption rates for monosaccharides *k*_*m_su*_, LCFAs (*k*__m_fa_), valerate and butyrate (*k*__m_c4_) had positive correlations with COD*_*eff*_*, whereas the half-saturation constants for amino acids (*K*__S_aa_), LCFAs (*K*__S_fa_), valerate and butyrate (*K*__S_c4_) had relatively weak negative correlations.

As shown in Figures 2D–F, *k*__m_ac_ had a strong negative correlation with the biogas flow rate in the AD system, whereas *k*__*m*_*pro*_ and *K*__S_ac_ had positive correlations. However, *K*__S_pro_ and *K*__S_h2_ had a negative correlation.

Thus, *k*__m_pro_, *K*__S_pro_, *k*__m_ac_, *K*__S_ac_, *k*__m_h2_, and *K*__S_h2_ were selected as parameters for estimating the model’s outputs to assess the COD*_*eff*_* and biogas production. Meanwhile, other 12 kinetic parameters were directly used the recommendation values by Batstone et al. (2002), which showed few correlation to the COD*_*eff*_* and biogas production modeling result by sensitivity analysis evaluation.

### Parameter Value Estimation

The sum of the squared errors (SSE) minimum function was applied for parameter estimation. SSE is also known as the “residual sum of squares” or “sum of squared residuals,” and it is the sum of the squares of the residuals in statistics (a measure of the deviations between the actual data and estimated model values). A small SSE value shows that the result obtained by the model agrees closely with the actual measured data. Using the on-site COD*_*eff*_* and biogas production data acquired from the anaerobic biochemical system for 150 days, the parameters were estimated using SSE fitting method. The results estimated for *k*__m_pro_, *K*__S_pro_, *k*__m_ac_, *K*__S_ac_, *k*__m_h2_, and *K*__S_h2_ are shown in Table 2.

**TABLE 2:** Parameter estimation result of the model.

	*k*_m_pro_	*K*_s_pro_	*k*_m_ac_	*K*_s_ac_	*k*_m_h2_	*K*_s_h2_	Relative sum of squares
Recommend values	13.0	0.3	8.0	0.15	35.0	2.5 × 10^–5^	–
Estimated values	12.02	0.35	4.26	0.26	16.62	3.21 × 10^–5^	5.06 × 10^–4^

Table 2 shows that the estimated values of *k*__m_pro_ and *K*__S_pro_ were close to the values recommended by IWA for high rate reactor on medium temperature, which indicates the system was not affected greatly by propionate absorption. However, the estimated values of *k*__m_ac_ and *k*__m_h2_ were about half of the values recommended by IWA, which shows that the absorption rates of *k*__m_ac_ and *k*__m_h2_ were relatively low in this system, and the corresponding half-saturation constants of *K*__S_ac_ and *K*__S_h2_ were slightly high. The parameter value estimation was based on the components of influent DIP wastewater. Due to the influent COD classification, the main components of the SCOD was monosaccharide and VFAs, and the ssCOD was carbohydrate. Further, the VFAs was simplified as S_ac_. The decomposition products from monosaccharide and carbohydrate were majorly S_ac_ and S_h2_. Because of that, the uptaking of S_ac_ and S_h2_ are the major factors of COD removing and methane producing. The high generation of S_ac_ and S_h2_ might lead to the difficulty of bacteria uptaking and removing, which resulted in the relatively low absorption rates and slightly high half-saturation constants of *k*__m_ac_ and *k*__m_h2_.

### Model Simulation and Validation

This anaerobic biochemical system treating DIP wastewater comprised an anaerobic pre-acidification tank and IC reactor. Based on the discussion above, several sensitive parameters in the simulation model were optimized using the estimated values presented in Table 2.

**FIGURE 3:**
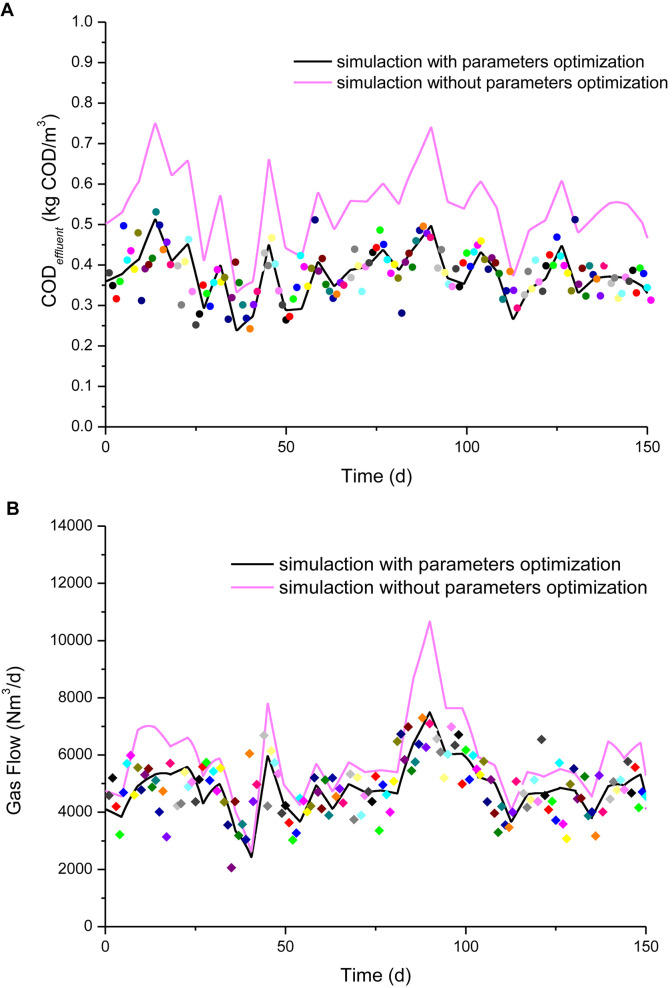
Schematic of comparing simulation result with measured values (color oblique square dot) for IC reactors using 150 days estimation period. **(A)** Simulation comparison of COD*_*eff*_*. **(B)** Simulation comparison of biogas production flow.

**FIGURE 4:**
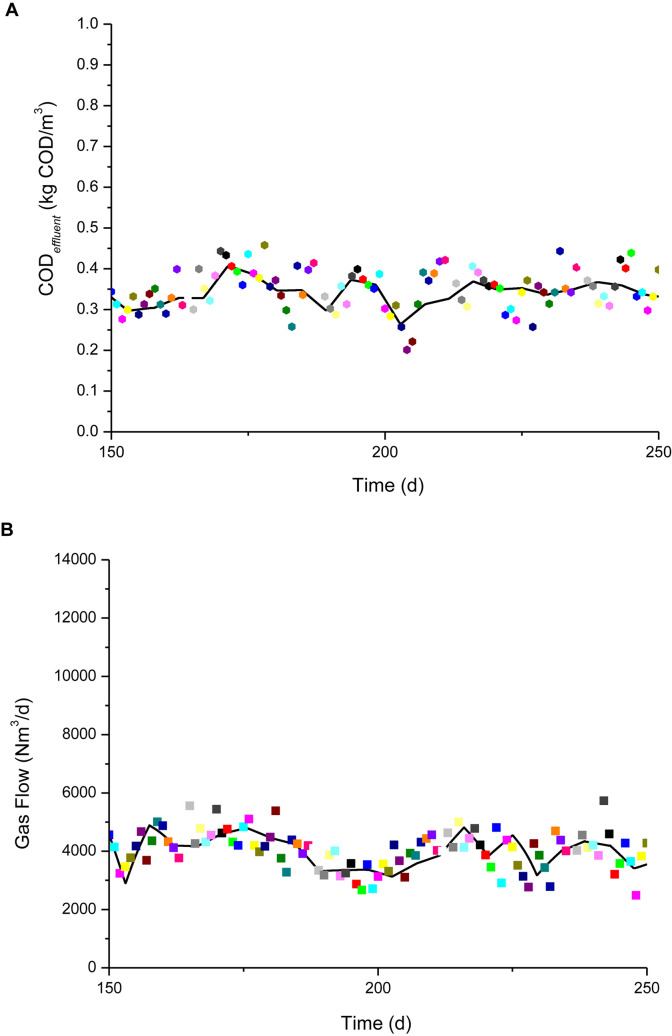
Schematic of simulation result with measured values (color oblique square dot) for IC reactors using subsequent 100-days operation period. **(A)** Simulation comparison of COD*_*eff*_*. **(B)** Simulation comparison of biogas production flow.

We compared the optimized parameters with the original parameters to simulate the results obtained from this anaerobic system lasting 150 days, as shown in Figure 3. Seeing from Figure 3A, after optimizing the parameters, the COD*_*eff*_* simulating result fits much better to the data (values) acquired from IC reactor on-site, comparing with those using recommended parameters. Similar results were obtained in the simulation of IC reactor biogas production, as shown in Figure 4B.

COD*_*eff*_* and biogas production data (values) were also acquired on-site from this anaerobic system over the following 100 days. These data were employed to validate the model and the simulation results are shown in Figure 4. The simulation results agreed well with the measured values. We concluded that the simulation results obtained using the optimized parameters were consistent with the actual COD*_*eff*_* and biogas production values for the anaerobic system.

According to global sensitivity analysis by the Monte Carlo technique, the model developed with the optimized parameters was more suitable for modeling anaerobic biochemical treatment in the DIP wastewater plant. In contrast to lab-scale experiments, the influent DIP wastewater could not be manipulated accurately by adjusting the inflow rate in the actual plant, especially the components of the experimental water. The composition of the raw product used in the paper and pulp mill was relatively simple, where it mainly comprised cellulose from wastepaper. The manufacturing technique and production process were unique and almost constant. Thus, the composition of the influent DIP wastewater was roughly stable in the first calibration period of 150 days and the subsequent validation period of 100 days, without major fluctuations. So, the parameters optimized based on the first 150 days were still suitable for modeling COD*_*eff*_* and biogas production by the IC reactor in the subsequent 100 days.

To further illustrate the advantages and disadvantages of the simulation model, the linear regression method was applied to quantitatively evaluate the accuracy of the simulation results. Linear fitting was performed between the predicted and measured values. The measured values were plotted on the *X*-axis and the predicted values on the *Y*-axis, and scatter plots were prepared. The slope of the fitted curve was set to 1, and the intercept was 0. The scatter plots will be distributed on or near the curve when the predicted and measured values are the same or similar.

Supplementary Figures 4, 5 show the linear fits of the measured and simulated COD*_*eff*_* and biogas flow values during the model parameter estimation period and the subsequent validation period, respectively. The results obtained by statistical fitting curve showed that the values simulated with the optimized parameters were evenly distributed on both sides of the fitted curve in the validation period (first 150 days) and validation period (second 100 days). By contrast, the values simulated using the recommended parameters clearly deviated from the fitted curve, where they were distributed above the curve, thereby demonstrating that the simulated values were much overestimated.

### Simulation Results for Pre-acidification Tank Effluent

In this complete anaerobic biochemical system used for treating DIP wastewater, the pre-acidification tank is connected before the IC reactor. In the simulation, the pre-acidification tank and IC reactor were run simultaneously as a combined module using Matlab 2017b/Simulink. According to the settings in the simulation of the pre-acidification tank described above, methanogenesis does not occur in the pre-acidification tank model (Supplementary Figure 2). Thus, the entire ADM1 was applied for simulating the pre-acidification tank except for the equation describing the methanogenesis process.

As described above, the pre-acidification tank is part of the AD treatment system and its simulation was also included in the overall sensitivity analysis. Therefore, it was feasible and necessary to study the simulation outputs in terms of COD*_*eff*_* and biogas production from the pre-acidification tank.

**FIGURE 5:**
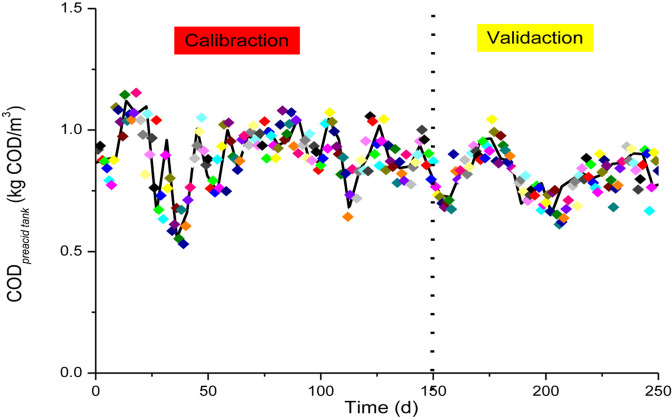
COD*_*eff*_* simulation curve and actual measured value of the effluent from the pre-acidification tank. Calibration part was in 150-days parameter estimation period, and validation part was in subsequent 100-days validation period.

From theoretical perspectives, under normal conditions, COD reduction and methane production do not occur in the wastewater pre-acidification process because of the short SRT. Figure 5 and Supplementary Figure 6 respectively show the simulation results in terms of COD*_*eff*_* and biogas production for the pre-acidification tank using the optimized parameters under real-time operating conditions. The results showed that the pre-acidification COD*_*eff*_* (COD*_*preacid tank*_*) obtained by the simulation was fairly consistent with the real measurements determined by sampling the pre-acidification tank effluent. In addition, the biogas production amount obtained by the simulation was maintained at zero. Therefore, the pre-acidification simulation results obtained by the model agreed well with the actual situation for the pre-acidification tank.

## Conclusion

The model established in this study was effective at simulating the effluent COD and biogas production by a DIP wastewater treatment anaerobic biochemical system. The proposed model is based on ADM1, and it also considers the hydrodynamics of the pre-acidification tank and full-scale IC reactor. The component interpretation method was used to separate and classify the influent components according to the requirements of ADM1. Global sensitivity analysis showed that the Monod absorption rates and half-saturation constants for propionic acid, acetic acid, and hydrogen were the most sensitive parameters, which were optimized further. The modeling results of calibration and validation were both in good agreement with the on-site COD*_*eff*_* and biogas production data. The effluent of pre-acidification tank was also fit well. The method proposed in this study may be useful for the design, operation, or monitoring of wastewater full-scale anaerobic reactors.

## Data Availability Statement

The original contributions presented in the study are included in the article/Supplementary Material, further inquiries can be directed to the corresponding author/s.

## Author Contributions

YH: methodology, investigation, software, and writing – original draft. YM: resources, supervision, conceptualization, and data curation. JW: supervision, finance supporting, and writing – review and editing. YW: writing – review and editing. All authors contributed to the article and approved the submitted version.

## Conflict of Interest

The authors declare that the research was conducted in the absence of any commercial or financial relationships that could be construed as a potential conflict of interest.

## Publisher’s Note

All claims expressed in this article are solely those of the authors and do not necessarily represent those of their affiliated organizations, or those of the publisher, the editors and the reviewers. Any product that may be evaluated in this article, or claim that may be made by its manufacturer, is not guaranteed or endorsed by the publisher.
