# Supplementary material for: Modeling the Performance of Full-Scale Anaerobic Biochemical System Treating Deinking Pulp Wastewater Based on Modified Anaerobic Digestion Model No. 1
Source: Front Microbiol. 2021 Sep 21;12:755398. doi: 10.3389/fmicb.2021.755398 (PMC8490887; doi:10.3389/fmicb.2021.755398)
Supplement: Supplementary file 1 [file Data_Sheet_1.docx]

# Figures


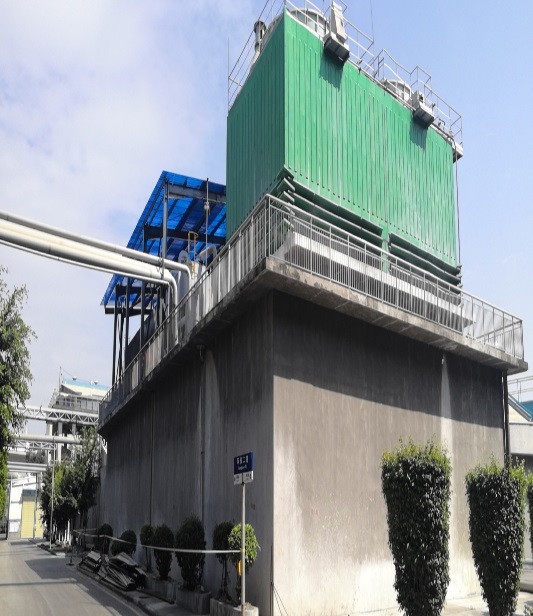

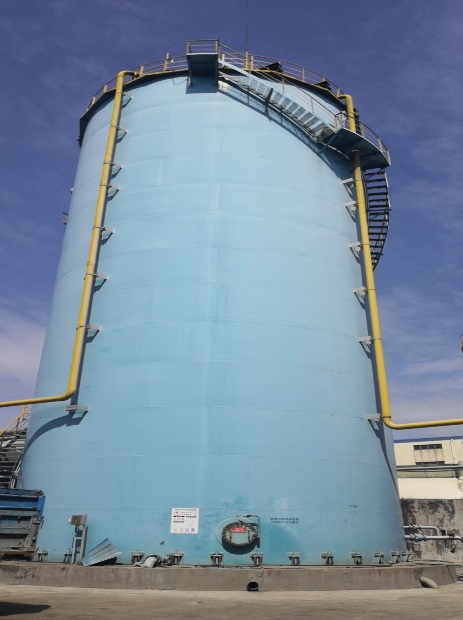


**Supplementary Figure 1.** The anaerobic digestion system for treating DIP wastewater is located in Guangzhou City (Guangdong Province, China). The top part is an anaerobic pre-acidification tank, and the under part is a full-scale IC reactor.

**Supplementary Figure 2.** Anaerobic biochemical processes of anaerobic pre-acidification and full-scale IC reactor by implement of ADM1.

**Supplementary Figure 3.** The influent composition COD distribution. (A) Proportion of suspended solid COD and soluble COD in influence; (B) Soluble components distributing proportion in soluble COD

**Supplementary Figure 4.** Linear fitting between the actual measured value and the simulated value of IC reactor COD*_eff_*, during the model parameter, 150-days estimation period (A-1 & B-1) and the subsequent 100-days validation period (A-2 & B-2).

**Supplementary Figure 5.** Linear fitting between the measured value and the simulated value of IC reactor biogas flow rate, during the model parameter 150 days estimation period (A-1 & B-1) and the subsequent 100 days validation period (A-2 & B-2).

**Supplementary Figure 6.** Simulation curve of biogas production in pre-acidification tank. Calibration part was in 150 days parameter estimation period, and Validation part was in subsequent 100 days validation period.

# Table

**Supplementary Table 1**

Main quality indicators of raw DIP wastewater.

| **Maximum water flow**  **m^3^ d**^−^**^1^** | **COD**  **mg L**^−^**^1^** | **BOD_5_**  **mg L**^−^**^1^** | **SS**  **mg L**^−^**^1^** | **pH** | **Temperature**  **℃** |
| --- | --- | --- | --- | --- | --- |
| 2 × 10^4^ | 2000~3000 | 1000~1500 | 1000~1500 | 6~9 | 55 |

**Supplementary Table 2**

Design of effluent quality indicator of AD treatment system.

| **COD**  **mg L**^−^**^1^** | **BOD_5_**  **mg L**^−^**^1^** | **SS**  **mg L**^−^**^1^** | **pH** | **Temperature**  **℃** |
| --- | --- | --- | --- | --- |
| 600~1050 | 300~500 | 100~200 | 6~8 | 30~35 |

# Supporting information

**Supplementary SI 1**

The classification of the inflow components when ADM1 is applied at actual scale or experimental scale mainly involves the following methods. (1) [Copp et al. (2003)](#_ENREF_3)and [Nopens et al. (2009)](#_ENREF_7) developed a model interface for connecting the Activated Sludge Model (ASM) output to the ADM1 input. However, [Buendia et al. (2008)](#_ENREF_2) showed that differences in biodegradation between aerobic and anaerobic conditions can lead to inconsistent models. (2) By referring to the oxygen uptake rate method from ASM, [Batstone et al. (2009)](#_ENREF_1) proposed a methane production rate (MPR) assay based on an appropriate inoculation test in a batch experiment. The MPR assay is relatively cheap to conduct, but it is time consuming and requires professional knowledge and experience. Moreover, the values obtained based on MPR measurements might be not applicable to dynamic simulation of a real full-scale reactor. (3) To eliminate the need for the MPR assay, anaerobic respirometry involves determining the influent COD components and other degradation-related kinetic parameters by interpreting the MPR curve ([Girault et al., 2012](#_ENREF_4); [Girault et al., 2011](#_ENREF_5)). However, anaerobic respirometry requires a large number of manual tests and it is difficult to apply in an actual plant. Differences in the ratio of the inoculum relative to that of the substrate can also greatly affect the test results. (4) According to the requirements for the ADM1 model input settings, the component interpretation method was proposed based on the detection of components such as proteins, lipids, carbohydrates, volatile fatty acids (VFAs), or fiber ([Wichern et al., 2009](#_ENREF_9); [Zaher et al., 2009](#_ENREF_10)). Appropriate estimates of the non-biodegradable inert components and hydrolysis rate constants should be made based on actual AD test data.

**Supplementary SI 2**

In terms of the anaerobic biological mechanism, during the acidogenesis and acetogenesis steps, the substrates are converted into intermediate fermentation products (mainly VFAs) by acid-producing bacteria. These intermediate products are further transformed into biogas in methanogenesis step by methanogenic bacteria. Previous studies have demonstrated that acid-producing bacteria have a rapid growth rate, where the generation time is generally 10~30 min. By contrast, methanogenic bacteria have a rather slow growth rate, where the generation time is up to 4~6 days ([Massey & Pohland, 1978](#_ENREF_6); [Sendjaja et al., 2015](#_ENREF_8)). Thus, by exploiting the obvious difference in the generation time, the HRT of the reactor can be manipulated to separate the acid-producing bacteria and methane-producing bacteria. Under a relatively short HRT, the methanogenic bacteria with a long generation time can be “washed out” of the reaction, whereas the acid-producing bacteria are selectively retained in the reactor.

Reference

Batstone, D., Tait, S., Starrenburg, D. 2009. Estimation of hydrolysis parameters in full-scale anerobic digesters. Biotechnology and bioengineering, 102(5), 1513-1520.

Buendia, I.M., Fernandez, F.J., Villasenor, J., Rodriguez, L. 2008. Biodegradability of meat industry wastes under anaerobic and aerobic conditions. Water Research, 42(14), 3767-3774.

Copp, J., Jeppsson, U., Rosen, C. 2003. Towards an ASM1 – ADM1 state variable interface for plant-wide wastewater treatment modeling.

Girault, R., Bridoux, G., Nauleau, F., Poullain, C., Buffet, J.-P., Steyer, J.P., Sadowski, A., Béline, F. 2012. A waste characterisation procedure for ADM1 implementation based on degradation kinetics. Water research, 46, 4099-4110.

Girault, R., Rousseau, P., Steyer, J.P., Bernet, N., Béline, F. 2011. Combination of batch experiments with continuous reactor data for ADM1 calibration: Application to anaerobic digestion of pig slurry. Water science and technology, 63(11), 2575-2582.

Massey, W., Pohland, F. 1978. Phase Separation of Anaerobic Stabilization by Kinetic Controls. Journal of the Water Pollution Control Federation, 50(1), 2204-2222.

Nopens, I., Batstone, D.J., Copp, J.B., Jeppsson, U., Volcke, E., Alex, J., Vanrolleghem, P.A. 2009. An ASM/ADM model interface for dynamic plant-wide simulation. Water Research, 43(7), 1913-1923.

Sendjaja, A.Y., Tan, Y., Pathak, S., Zhou, Y., bin Abdul Majid, M., Liu, J.L., Ng, W.J. 2015. Regression based state space adaptive model of two-phase anaerobic reactor. Chemosphere, 140, 159-166.

Wichern, M., Gehring, T., Fischer, K., Andrade, D., Lübken, M., Koch, K., Gronauer, A., Horn, H. 2009. Monofermentation of grass silage under mesophilic conditions: Measurements and mathematical modeling with ADM 1. Bioresource Technology, 100(4), 1675-1681.

Zaher, U., Li, R., Jeppsson, U., Steyer, J., Chen, S. 2009. GISCOD: General Integrated Solid Waste Co-Digestion model. Water Research, 43(10), 2717-2727.
